# Association of neighborhood‐level social determinants of health with psychosocial distress in patients newly diagnosed with lung cancer

**DOI:** 10.1002/cnr2.1734

**Published:** 2022-10-17

**Authors:** Oluwabunmi M. Emidio, Sarah L. Cutrona, Sharina D. Person, Kathleen M. Mazor, Christine Frisard, Stephenie C. Lemon

**Affiliations:** ^1^ Department of Population and Quantitative Health Sciences University of Massachusetts Chan Medical School Worcester Massachusetts USA; ^2^ Department of Medicine University of Massachusetts Chan Medical School Worcester Massachusetts USA

**Keywords:** lung cancer, psychosocial distress, social determinants of health

## Abstract

**Background and Aim:**

Patients with lung cancer experience high rates of psychosocial distress. They are also more likely to have unresolved, unmet social needs which may contribute to psychosocial distress. Despite this, neighborhood‐level social determinants of health (SDOH) in relation to psychosocial distress have not been adequately investigated in patients with lung cancer. The goal of this study is to examine the association between neighborhood‐level SDOH and psychosocial distress among a sample of newly diagnosed patients with lung cancer.

**Methods:**

This cross‐sectional study included newly diagnosed, adult lung cancer patients from an accredited cancer center. Psychosocial distress was measured with the Distress Thermometer. Neighborhood‐level SDOH indicators for income and education were used to create a composite SDOH variable categorized into low, medium, and high deprivation levels. Covariates were age, gender, race/ethnicity, comorbidity index, cancer stage, and insurance status. Using multivariate logistic regression modeling, the association of psychosocial distress with the neighborhood‐level SDOH was examined.

**Results:**

The prevalence of psychosocial distress in the sample was 58.4%. Neighborhood‐level SDOH indicators were not significantly associated with psychosocial distress. Higher odds of psychosocial distress were significantly associated with being female and having distant or regional cancer versus localized cancer. The age group 66–75 years was significantly associated with lower distress compared with those aged <65 years.

**Conclusions:**

Psychosocial distress was consistently high across all the SDOH deprivation categories; but these neighborhood‐level SDOH indicators do not appear to be predictive of psychosocial distress at the time of diagnosis of lung cancer.

## 1 INTRODUCTION

Lung cancer is the second most frequently diagnosed cancer and the leading cause of cancer death worldwide.^1^, ^2^ In 2020, there were an estimated 2.2 million new cases and 1.8 million deaths from lung cancer globally.^1^ The American Cancer Society estimated that there would be approximately 235 760 new cases and 131 880 deaths of lung cancer in the US in 2021.^2^

Psychosocial distress as defined in the National Comprehensive Cancer Network (NCCN) clinical guidelines is “a multifactorial, unpleasant experience of a psychological (i.e., cognitive, behavioral, emotional), social, spiritual, and/or physical nature, that may interfere with the ability to cope effectively with cancer, its physical symptoms and its treatment”.^3^ Psychosocial distress is a widely recognized occurrence among patients with cancer.^4^ In response, the American College of Surgeons' Commission on Cancer enacted accreditation standards that require cancer centers to implement the five steps of comprehensive psychosocial distress screening, as a means to ensure that patients with distress are promptly identified and treated.^5^ The five steps include: screening to identify distress, evaluation to determine the cause of the distress, appropriate referral pathways initiated with follow‐up, and documentation with regular auditing for quality improvement.^6^

In comparison with other cancer populations, lung cancer patients have one of the highest rates of psychosocial distress, with reported rates ranging between 43.5% to 61.6%.^7^, ^8^, ^9^, ^10^ Factors that can contribute to this are poorer prognosis and increased burden of symptoms of the cancer^11^, ^12^ as well as stigma and self‐blame because of the causal impact of prolonged tobacco use.^11^, ^13^, ^14^ Consequences of psychosocial distress in cancer patients include difficulty in making treatment decisions with resulting delay in care, disease progression, negative health outcomes, reduced quality of life, and poorer prognosis.^15^, ^16^, ^17^ It is therefore important to identify which particular lung cancer patient groups are at high risk for psychosocial distress.

In various studies, several factors were found to be associated with higher levels of psychosocial distress^10^, ^18^, ^19^: younger age, female gender, current cigarette smoking, advanced stage of disease, receiving surgery or chemotherapy and a higher ECOG scale of performance status score. These studies were however limited to the examination of demographic and clinical factors. Examining social determinants of health (SDOH) in healthcare research is important because they influence health care outcomes and associated disparities in care delivery.^20^, ^21^ SDOH are “the conditions in the environments where people are born, live, learn, work, play, worship, and age that affect a wide range of health, functioning, and quality‐of‐life outcomes and risks”.^22^ The SDOH of a community often are rooted in its overall socioeconomic status (SES), as those with lower SES tend to live in areas with poor SDOH. SES indicators are considered in defining SDOH.^23^ SDOH associated with lower SES have been linked with greater unmet health needs (physical and social), poorer health outcomes, health inequities and psychosocial distress.^20^, ^21^, ^24^, ^25^ Low SES and psychosocial distress are often independently associated with poorer health outcomes.^3^, ^26^, ^27^ Studies examining the relationship between SES and psychological distress have reported a synergistic effect of worsened health outcomes in distressed patients with low SES.^28^, ^29^ High levels of psychological distress were most common among individuals with lower levels of SES and social capital, even after adjusting for participant demographic characteristics and life events.^30^ These studies were, however, conducted in non‐cancer populations. Despite the high prevalence of psychosocial distress^7^, ^8^, ^9^, ^10^ and high proportion of unmet social needs^31^, ^32^ in patients diagnosed with lung cancer and the correlation of these factors with SDOH, to the best of our knowledge, no study has investigated the association of SDOH and psychosocial distress in patients with lung cancer.

Therefore, the goal of this study was to assess the association between neighborhood‐level SDOH and psychosocial distress among newly diagnosed lung cancer patients. We hypothesized that newly diagnosed patients with lung cancer who experienced greater neighborhood‐level SDOH disadvantage (i.e., who live in a more socioeconomically deprived neighborhood) would experience higher levels of psychosocial distress. The formulation of this hypothesis was guided by elements of the Commission on Social Determinants of Health (CSDH) conceptual framework. This framework posits that indicators of socioeconomic position such as income and education operate through a set of intermediary determinants of health including neighborhood quality and psychosocial stressors, to shape health outcomes.

## 2 MATERIALS AND METHODS

### 2.1 Study design & setting

This study utilized a cross‐sectional study design with patient data from the UMass Memorial Health Care (UMMHC) System. UMMHC is the largest health care system in Central and Western Massachusetts. The Cancer Center in UMMHC is accredited by the American College of Surgeons Commission on Cancer (CoC).

### 2.2 Study sample

All newly diagnosed adult lung cancer patients seen within UMMHC between 2017 and 2021, and who completed a psychosocial distress screening were included in this study. A total of 1516 patients were newly diagnosed with lung cancer and of these, 1115 had no recorded data on psychosocial distress scores, thus they were excluded. This resulted in an analytic sample size of 401.

### 2.3 Data sources

Data were obtained from the University of Massachusetts Chan Medical School (UMMS) Research Informatics Core. UMMS is the academic partner of UMMHC. The Research Informatics Core provides access to and supports the use of multiple and complex data sources. For this study, the data sources included (1) Electronic Medical Records which were the data source for the sociodemographic, clinical and psychosocial distress data, and (2) Census data from the 2010 5‐year ACS survey.^33^ Participants' addresses were geocoded to obtain corresponding census tract information, which provides neighborhood‐level indices of SDOH. A manual chart review and data abstraction of the electronic medical records captured in EPIC were also conducted to obtain missing data and to clarify ambiguous data.

### 2.4 Ethical approval

Approval for the study and waiver of consent were received from the UMMS Committee for the Protection of Human Subjects in Research.

## 3 MEASURES

### 3.1 Psychosocial distress

The psychosocial distress data are obtained from the participants using the Distress Thermometer (DT) and its associated problem list.^34^ In UMMHC, the DT is completed by patients at the time of the first in‐person visit following a cancer diagnosis. The DT is a validated, single‐item patient‐reported visual scale developed by the National Comprehensive Cancer Network (NCCN).^34^ It has a range of 0–10, with 0 indicating no distress and 10 indicating extreme distress. Patients are asked to circle the number that best describes how much distress they have experienced over the past week. A suggested cut‐off value for the DT to identify distress by the NCCN is 4.^34^, ^35^ Studies have been done to describe the translation and validation of the DT in cancer patients across different cultures and countries. In the majority of examined studies, a cut point of 4 to indicate significant level of distress, maximized sensitivity and specificity, when compared to a well‐established benchmark such as the Hospital Anxiety and Depression Scale (HADS).^36^, ^37^, ^38^, ^39^, ^40^ In this study, we therefore categorized patients reporting a distress level of 4 or greater as distressed, and those reporting a distress level less than 4 as non‐distressed.

### 3.2 Neighborhood‐level social determinants of health

Neighborhood‐level SDOH data were obtained by geocoding the participants' address at time of diagnosis, to obtain census tract‐level information. We chose standard economic indicators with known associations with health outcomes.^41^, ^42^, ^43^ These indicators and operational definitions are neighborhood income (aggregated from census tract median household income), and neighborhood education (aggregated from the census tract percent of high school graduates). Each indicator's sample mean value was used as a cutoff and we grouped patients with a value equal to or greater than the mean as living in a neighborhood with SDOH with low deprivation level and those reporting a value less than the mean as living in a neighborhood with high deprivation level SDOH.

**FIGURE 1:**
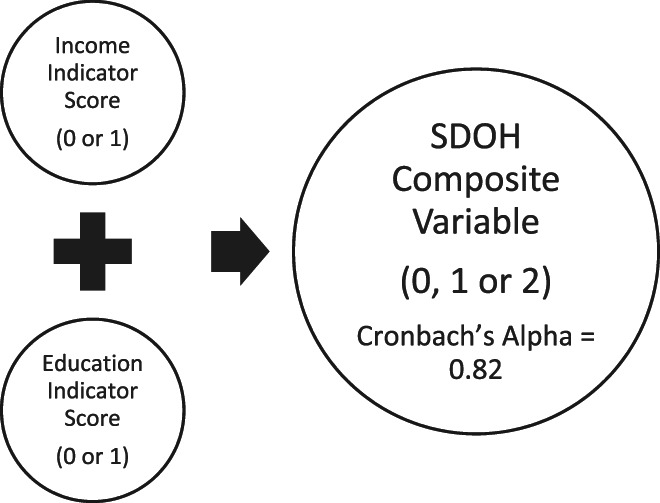
Composite SDOH variable

To address multicollinearity of these indicators,^44^ a composite SDOH variable was created by summing the two individual indicators. Each indicator had a score of 0 if low deprived SDOH or 1 if high deprived SDOH. Therefore, the composite SDOH variable had a score range of 0 to 2 and a higher score meant greater SDOH deprivation. This variable was labeled as low, medium, and high neighborhood‐level SDOH deprivation levels (Figure 1), allowing us to compare individuals with higher level of neighborhood‐level SDOH deprivation to those with lower levels.

### 3.3 Covariates

A priori choice of covariates was made based on factors found in the literature to be associated with psychosocial distress in lung cancer patients.^10^, ^18^, ^19^ Sociodemographic and clinical information collected were age at diagnosis (≤65, 66–75, >75), gender, race/ethnicity (Hispanic/Latino, Non‐Hispanic White, Non‐Hispanic Others), cancer stage at diagnosis (stage I/localized, stage II & III/regional, stage IV/distant), insurance (Medicare/MassHealth/Private) and comorbidity disease count (0, 1, ≥2). We carefully curated a list of comorbid conditions that are often highly prevalent in lung cancer and are known to have impactful interaction with lung cancer.^45^, ^46^ For the comorbidity count for each patient, we counted the number of these conditions that were present in the electronic health records using ICD‐10 codes.^47^ The selected comorbid conditions included myocardial infarction, hypertension, congestive heart failure, peripheral vascular disease, cerebrovascular disease, COPD, diabetes, renal disease, liver disease, and rheumatologic disease.

## 4 STATISTICAL ANALYSIS

Descriptive analysis (counts and proportions) was calculated overall and by distress levels to describe the characteristics of all variables. Pearson's chi‐square tests were used to evaluate the bivariate relationship between the psychosocial distress outcome and the demographic, clinical, and neighborhood‐level SDOH variables. *p* value < .05 was considered significant. Logistic regression models were used to predict the associations of clinically significant distress, defined as a psychosocial distress score of ≥4. We first calculated the unadjusted odds ratios and 95% Confidence Interval (CI), then a fully adjusted model controlling for the sociodemographic and clinical variables. All statistical analyses were performed using Stata 17.0 statistical software (StataCorp, College Station, TX).

## 5 RESULTS

### 5.1 Sample description

Approximately one‐third of the 401 study participants were aged ≤65 years (33.9%), and 52% were female. Most participants (91%) were non‐Hispanic White. One‐quarter (25%) had localized (stage I) cancer. More than 80% of participants had at least one comorbid condition. All participants had a form of insurance and the majority (82%) had public insurance.

The mean neighborhood‐level SDOH indicator values were $65 000 for median household income, and 88% for percentage of high school graduates. With respect to the neighborhood‐level SDOH composite variable, almost half (48.7%) of the participants were classified in the low deprivation category. (Table 1).

**TABLE 1:** Prevalence of psychosocial distress by sample characteristics

	Total		No psychosocial distress (Score < 4)^b^		Psychosocial distress (Score ≥ 4)^b^		*p* Value
	*N*	%	*N*	%	*N*	%	
Overall	401	100	167	41.6	234	58.4	
Gender							<.001
Female	208	51.9	58	27.9	150	72.1	
Male	193	48.1	109	56.5	84	43.5	
Age (years)							.033
≤65	136	33.9	46	33.8	90	66.2	
66–75	142	35.4	70	49.3	72	50.7	
≥76	123	30.7	51	41.5	72	58.5	
Race/Ethnicity^a^							.618
Hispanic/Latino	17	4.3	9	52.9	8	47.1	
Non‐Hispanic white	363	91.2	150	41.3	213	58.7	
Non‐Hispanic other	18	4.5	7	38.9	11	61.1	
Stage at diagnosis							.008
Localized	101	25.2	54	53.5	47	46.5	
Regional	157	39.2	65	41.4	92	58.6	
Distant	143	35.7	48	33.6	95	66.4	
Comorbid disease score							.917
0	71	17.7	28	39.4	43	60.6	
1	114	28.4	48	42.1	66	57.9	
≥2	216	53.9	91	42.1	125	57.9	
Health insurance^a^							.137
Medicare	275	68.8	123	44.7	152	55.3	
Mass health	53	13.2	17	32.1	36	67.9	
Private	72	18.0	26	36.1	46	63.9	
SDOH composite variable^a^ (Deprivation level)							
Low	151	48.7	63	41.7	88	58.3	.388
Medium	48	15.5	16	33.3	32	66.7	
High	111	35.8	50	45.0	61	55.0	

^a^ Missing data on ethnicity (*n* = 3), health insurance (*n* = 1), SDOH (*n* = 91).

^b^ Distress scores range from 1–10.

### 5.2 Prevalence of psychosocial distress

Of the 401 participants included in the study sample, 234 (58.4%) had distress score of ≥4. The prevalence of distress was significantly higher in participants who were female (72.1%), those younger than 66 years (66.2%), and those with distant (stage IV) cancer (66.4%) (Table 1).

### 5.3 Logistic regression analysis

The neighborhood‐level SDOH composite variable was not significantly associated with psychosocial distress even after fully adjusting for other a priori‐defined covariates (OR_medium_ = 1.27, 95% CI = 0.60; 2.69), (OR_high_ = 0.78, 95% CI = 0.44; 1.39; Table 2). Using a fully adjusted model, female participants had a 3.97 times higher likelihood of psychosocial distress compared to male patients (OR = 3.97, 95% CI = 2.39; 6.60). Participants aged 66–75 years had 57% lower likelihood of psychosocial distress compared with those aged below 65 years (OR = 0.43, 95% CI = 0.19; 0.95). Having regional or distant stage cancer was significantly associated with higher risk of psychological distress, compared to those with localized (stage I) cancer (OR_reg_ = 1.91, 95% CI = 1.01; 3.62), (OR_distant_ = 2.56, 95% CI = 1.31; 5.01) (Table 2).

**TABLE 2:** Unadjusted and fully adjusted odds ratios (ORs) of psychosocial distress by participants' characteristics

	Unadjusted OR (95%CI)	Adjusted OR (95%CI)^a^
Gender		
Male	Ref	Ref
Female	3.36 (2.21–5.09)	3.97 (2.39–6.60)
Age (years)		
≤65	Ref	Ref
66–75	0.53 (0.32–0.85)	0.43 (0.19–0.95)
≥76	0.72 (0.44–1.20)	0.55 (0.23–1.30)
Race/Ethnicity		
Non‐Hispanic white	Ref	Ref
Non‐Hispanic other	1.11 (0.42–2.92)	0.80 (0.26–2.46)
Hispanic/Latino	0.63 (0.24–1.66)	0.32 (0.09–1.12)
Stage at diagnosis		
Localized	Ref	Ref
Regional	1.63 (0.98–2.69)	1.91 (1.01–3.62)
Distant	2.27 (1.35–3.84)	2.56 (1.31–5.01)
Comorbid disease score		
0	Ref	Ref
1	0.90 (0.49–1.64)	1.16 (0.52–2.57)
≥2	0.89 (0.52–1.55)	0.94 (0.44–1.97)
Health insurance		
Medicare	Ref	Ref
MassHealth	1.71 (0.92–3.20)	1.0 (0.38–2.63)
Private	1.43 (0.84–2.45)	0.69 (0.29–1.66)
SDOH composite variable (Deprivation level)		
Low	Ref	Ref
Medium	1.43 (0.72–2.83)	1.27 (0.60–2.69)
High	0.87 (0.53–1.43)	0.78 (0.44–1.39)

^**a**^ Model fully adjusted for all listed variables.

## 6 DISCUSSION

The study was to our best knowledge the first to examine the relationship between neighborhood‐level SDOH and psychosocial distress in newly diagnosed lung cancer patients. Our study findings, both in the univariate and multivariate analyses, showed no association between neighborhood‐level SDOH and psychosocial distress levels. This result was inconsistent with our initial hypothesis. We expected that those participants with SDOH reflective of high deprivation levels would also have an increased probability of suffering from distress at the time of lung cancer diagnosis. Instead, we found a high proportion of clinically meaningful psychosocial distress across all SDOH deprivation levels, suggesting that the diagnosis of lung cancer may generate distress irrespective of the patient's neighborhood socioeconomic circumstances. This overall high proportion of patients with elevated psychosocial distress in the study sample (58.4%) is consistent with previous studies in lung cancer patients.^7^, ^8^, ^9^, ^10^ While cancer centers are required to implement psychosocial screening for accreditation,^5^ optimal timing of screening and referral practices remain challenges. The trajectory of psychosocial distress after initial diagnosis remains unknown, as do population subgroup differences. In this study, psychosocial distress was ascertained only one time, at the first visit to the cancer center following diagnosis, which is the procedure of UMMHC. Therefore, we were unable to assess if the high level of psychosocial distress observed in this sample persisted throughout the course of treatment nor could we assess the association of neighborhood‐level SDOH with psychosocial distress levels across different time points in the cancer care continuum.

An important observation was the high proportion of newly diagnosed lung cancer patients who did not have documented psychosocial distress scores. Only about 26% had documented distress‐screening scores. There did not appear to be differences in the characteristics of those who had documented distress scores and those who did not. Like the study sample, most of the patients without distress scores were non‐Hispanic White (89%), 52% were female and majority had public insurance. This lack of documentation may preclude adequate monitoring, evaluation, and follow‐up of distressed patients.

We observed differences in psychosocial distress by gender, age group, and cancer stage. Being female and having regional/distant disease compared to localized disease were significantly associated with higher likelihood of being distressed. In comparison with those aged 65 years or younger, participants aged 66 to 75 years were less likely to report psychosocial distress. These results are consistent with the literature on factors associated with psychosocial distress among lung cancer patients.^18^, ^19^ Our findings reinforce the need for lung cancer care centers to provide resources to meet the psychosocial needs of patients.

The strengths of this study include its innovation as the first to evaluate the association of SDOH and psychosocial distress in lung cancer patients. Other innovations that strengthen this study are the creation of a SDOH composite variable for better analysis and a comorbid disease score comprising a carefully curated list of comorbid conditions prevalent in lung cancer patients to reduce potential confounding. Composite variables help reduce the incidence of Type I error and multicollinearity for regression analysis.^44^

### 6.1 Study limitations

An important limitation was that only about 26% of the newly diagnosed patients with lung cancer had documented distress‐screening scores, thus reducing the sample size. The cross‐sectional study design precludes assessment of causation. Another limitation is assessment of SDOH and psychosocial distress at only one time, which precludes assessing if the absence of an association is consistent across different time points in the cancer continuum. Also, in this study, out of the five key domains of SDOH–economic stability, education access and quality, health care access and quality, neighborhood and built environment, social and community context,^48^ we could only access measures for economic stability and education. Lastly, participants were from only one cancer care center, which limits generalizability. However, the median values observed on the three SDOH indicators were very similar to national averages, indicating that our sample is somewhat reflective of the US population.

### 6.2 Clinical implication

Findings from this study emphasize the importance of comprehensive psychosocial distress screening which includes the prompt identification, management, and proper documentation of psychosocial distress, in all lung cancer patients regardless of any other patient characteristics. Ensuring that all patients are screened irrespective of their SODH deprivation level will likely improve equitable whole‐person care.

## 7 CONCLUSION

Psychosocial distress was consistently high overall and with no variation by neighborhood‐level SDOH deprivation levels, in this sample of patients newly diagnosed with lung cancer. This suggests that the diagnosis of lung cancer may by itself; generate psychosocial distress irrespective of the patient's neighborhood socioeconomic circumstances. Therefore, ensuring that comprehensive psychosocial distress screening is implemented for all lung cancer patients is critical. Future research exploring the association of SDOH with psychosocial distress post‐diagnosis may be warranted because SDOH which strongly influence health related behaviors and are known drivers of disparities in healthcare,^49^ may have a significant association with psychosocial distress at different time points across the lung cancer care continuum.

## AUTHOR CONTRIBUTIONS

**Oluwabunmi M. Emidio:** Conceptualization (lead); data curation (lead); formal analysis (lead); investigation (lead); methodology (lead); project administration (lead); writing – original draft (lead); writing – review and editing (lead). **Christine Frisard:** Data curation (supporting); formal analysis (supporting); software (supporting). **Sarah L. Cutrona:** Conceptualization (supporting); methodology (supporting); resources (supporting); writing – review and editing (supporting). **Kathleen M. Mazor:** Conceptualization (supporting); methodology (supporting); supervision (supporting); writing – review and editing (supporting). **Sharina D. Person:** Conceptualization (supporting); data curation (supporting); formal analysis (supporting); methodology (supporting); writing – review and editing (supporting). **Stephenie C. Lemon:** Conceptualization (equal); data curation (supporting); methodology (equal); resources (lead); supervision (lead); writing – review and editing (lead).

## ETHICAL STATEMENT

Approval for the study and waiver of consent were received from the UMMS Committee for the Protection of Human Subjects in Research.

## FUNDING INFORMATION

The Prevention and Control of Cancer: Training in Implementation Science (PRACCTIS) Program (National Cancer Institute Grant # T32 CA172009).

## CONFLICT OF INTEREST

The authors declare no conflicts of interest.

## Data Availability

The data that support the findings of this study are available from the corresponding author upon reasonable request.

## References

[1] Sung H , Ferlay J , Siegel RL , et al. Global cancer statistics 2020: GLOBOCAN estimates of incidence and mortality worldwide for 36 cancers in 185 countries. CA Cancer J Clin. 2021;71(3):209‐249. 33538338 10.3322/caac.21660

[2] Siegel RL , Miller KD , Fuchs HE , Jemal A . Cancer statistics, 2021. CA Cancer J Clin. 2021;71(1):7‐33. 33433946 10.3322/caac.21654

[3] Riba MB , Donovan KA , Andersen B , et al. Distress management, version 3.2019, NCCN clinical practice guidelines. Oncology. 2019;17(10):1229. 10.6004/jnccn.2019.0048 PMC6907687 31590149

[4] Gorman L . The psychosocial impact of cancer on the individual, family, and society. Psychosocial Nursing Care along the Cancer Continuum. Oncology Nursing Society; 1998:3‐25.

[5] Cancer ACoSCo . Cancer Program Standards: Ensuring Patient‐Centered Care. 2016. 2019.

[6] Lazenby M , Tan H , Pasacreta N , Ercolano E , McCorkle R . The five steps of comprehensive psychosocial distress screening. Curr Oncol Rep. 2015;17(5):447. 25824699 10.1007/s11912-015-0447-z PMC4918509

[7] Badger T , Lebaron VT , Mccorkle R . Screening for emotional distress in older patients with lung cancer. Lung Cancer. 2010;24(4).

[8] Carlson LE , Zelinski EL , Toivonen KI , et al. Prevalence of psychosocial distress in cancer patients across 55 north American cancer centers. J Psychosoc Oncol. 2019;37(1):5‐21. 30592249 10.1080/07347332.2018.1521490

[9] Zabora J , Brintzenhofeszoc K , Jacobsen P , et al. A new psychosocial screening instrument for use with cancer patients. Psychosomatics. 2001;42(3):241‐246. 11351113 10.1176/appi.psy.42.3.241

[10] Graves KD , Arnold SM , Love CL , Kirsh KL , Moore PG , Passik SD . Distress screening in a multidisciplinary lung cancer clinic: prevalence and predictors of clinically significant distress. Lung Cancer (Amsterdam, Netherlands). 2007;55(2):215‐224. 10.1016/j.lungcan.2006.10.001 PMC1857305 17084483

[11] Lehto RH . Psychosocial challenges for patients with advanced lung cancer: interventions to improve well‐being. Lung Cancer (Auckl). 2017;8:79‐90. 28814906 10.2147/LCTT.S120215 PMC5546827

[12] Ferrell B , Sun V , Hurria A , et al. Interdisciplinary palliative Care for Patients with Lung Cancer. J Pain Symptom Manage. 2015;50(6):758‐767. 26296261 10.1016/j.jpainsymman.2015.07.005 PMC4666729

[13] Carolyn Messner D , LCSW‐R B. Lung Cancers, and Stigma: Perception or Reality?.

[14] Chapple A , Ziebland S , McPherson A . Stigma, shame, and blame experienced by patients with lung cancer: qualitative study. BMJ. 2004;328(7454):1470. 15194599 10.1136/bmj.38111.639734.7C PMC428516

[15] Vodermaier A , Lucas S , Linden W , Olson R . Anxiety after diagnosis predicts lung cancer‐specific and overall survival in patients with stage III non‐small cell lung cancer: a population‐based cohort study. J Pain Symptom Manage. 2017;53(6):1057‐1065. 28063862 10.1016/j.jpainsymman.2016.12.338

[16] Barrera I , Spiegel D . Review of psychotherapeutic interventions on depression in cancer patients and their impact on disease progression. Int Rev Psychiatry. 2014;26(1):31‐43. 24716499 10.3109/09540261.2013.864259

[17] Holland JC , Andersen B , Breitbart WS , et al. Distress management: clinical practice guidelines in oncology™. JNCCN J Natl Compr Cancer Netw. 2007;5(1):66‐98. 17239328

[18] Morrison EJ , Novotny PJ , Sloan JA , et al. Emotional problems, quality of life, and symptom burden in patients with lung cancer. Clin Lung Cancer. 2017;18(5):497‐503. 28412094 10.1016/j.cllc.2017.02.008 PMC9062944

[19] Leung B , Laskin J , Wu J , Bates A , Ho C . Assessing the psychosocial needs of newly diagnosed patients with nonsmall cell lung cancer: identifying factors associated with distress. Psychooncology. 2019;28(4):815‐821. 30758101 10.1002/pon.5025

[20] Heiman HJ , Artiga S . Beyond Health Care: the Role of Social Determinants in Promoting Health and Health Equity. KFF.org; 2015.

[21] Solar O , Irwin A . A Conceptual Framework for Action on the Social Determinants of Health. WHO Document Production Services; 2010.

[22] Healthy People 2030 USDoHaHS, Office of Disease Prevention and Health Promotion . Retrieved September 15 2021, from https://health.gov/healthypeople/objectives-and-data/social-determinants-health.

[23] Prevention OoD, Promotion H. Healthy People 2020: Social determinants of health. Retrieved September 2020;5:2019.

[24] Alegría M , NeMoyer A , Falgàs Bagué I , Wang Y , Alvarez K . Social determinants of mental health: where we are and where we need to go. Curr Psychiatry Rep 2018;20(11):95‐, 20. 30221308 10.1007/s11920-018-0969-9 PMC6181118

[25] Cole MB , Nguyen KH . Unmet social needs among low‐income adults in the United States: associations with health care access and quality. Health Serv Res. 2020;55(S2):873‐882. 32880945 10.1111/1475-6773.13555 PMC7518813

[26] Flaskerud JH , DeLilly CR . Social determinants of health status. Issues Ment Health Nurs. 2012;33(7):494‐497. 22757603 10.3109/01612840.2012.662581 PMC3710744

[27] Taghizadeh A , Pourali L , Vaziri Z , Saedi HR , Behdani F , Amel R . Psychological distress in cancer patients. Middle East J Cancer. 2018;9(2):143‐149.

[28] Lazzarino AI , Hamer M , Stamatakis E , Steptoe A . The combined association of psychological distress and socioeconomic status with all‐cause mortality: a national cohort study. JAMA Intern Med. 2013;173(1):22‐27. 23212347 10.1001/2013.jamainternmed.951

[29] Pond E , Fowler K , Hesson J . The influence of socioeconomic status on psychological distress in Canadian adults with ADD/ADHD. J Atten Disord. 2019;23(9):940‐948. 27288904 10.1177/1087054716653214

[30] Myer L , Stein DJ , Grimsrud A , Seedat S , Williams DR . Social determinants of psychological distress in a nationally‐representative sample of south African adults. Soc Sci Med. 2008;66(8):1828‐1840. 18299167 10.1016/j.socscimed.2008.01.025 PMC3203636

[31] Giuliani M . MS17.01 unmet needs and QOL of lung cancer survivors. J Thorac Oncol. 2018;13(10):S276‐S277.

[32] Giuliani M , Milne R , Puts M , et al. The prevalence and nature of supportive care needs in lung cancer patients. Curr Oncol. 2016;23(4):258‐265. 27536176 10.3747/co.23.3012 PMC4974033

[33] Herman E . The American community survey: an introduction to the basics. Gov Inf Q. 2008;25(3):504‐519.

[34] Holland JC , Andersen B , Breitbart WS , et al. Distress management. J Natl Compr Cancer Netw. 2013;11(2):190‐209. 10.6004/jnccn.2013.0027 23411386

[35] Network NCC . NCCN Clinical Practice Guidelines in Oncology. Distress management. Version 1.2017. Fort Washington, PA. 2017.

[36] Ozalp E , Cankurtaran ES , Soygür H , Geyik PO , Jacobsen PB . Screening for psychological distress in Turkish cancer patients. Psychooncology. 2007;16(4):304‐311. 16909427 10.1002/pon.1059

[37] Shim EJ , Shin YW , Jeon HJ , Hahm BJ . Distress and its correlates in Korean cancer patients: pilot use of the distress thermometer and the problem list. Psychooncology. 2008;17(6):548‐555. 17957764 10.1002/pon.1275

[38] Tuinman MA , Gazendam‐Donofrio SM , Hoekstra‐Weebers JE . Screening and referral for psychosocial distress in oncologic practice: use of the distress thermometer. Cancer. 2008;113(4):870‐878. 18618581 10.1002/cncr.23622

[39] Donovan KA , Grassi L , McGinty HL , Jacobsen PB . Validation of the distress thermometer worldwide: state of the science. Psychooncology. 2014;23(3):241‐250. 25160838 10.1002/pon.3430

[40] Jacobsen PB , Donovan KA , Trask PC , et al. Screening for psychologic distress in ambulatory cancer patients: a multicenter evaluation of the distress thermometer. Cancer. 2005;103(7):1494‐1502. 15726544 10.1002/cncr.20940

[41] Chetty R , Stepner M , Abraham S , et al. The association between income and life expectancy in the United States, 2001–2014. JAMA. 2016;315(16):1750‐1766. 27063997 10.1001/jama.2016.4226 PMC4866586

[42] Krueger PM , Tran MK , Hummer RA , Chang VW . Mortality attributable to low levels of education in the United States. PLoS One. 2015;10(7):e0131809. 26153885 10.1371/journal.pone.0131809 PMC4496052

[43] Freeman AK . Racial residential segregation and the distribution of health‐related organizations in urban neighborhoods. Soc Probl. 2017;64(2):256‐276.

[44] Song M‐K , Lin F‐C , Ward SE , Fine JP . Composite variables: when and how. Nurs Res. 2013;62(1):45‐49. 23114795 10.1097/NNR.0b013e3182741948 PMC5459482

[45] Dutkowska A , Antczak A . Comorbidities in lung cancer. Advances in Respiratory Medicine. 2016;84(3):186‐192. 10.5603/PiAP.2016.0022 27238182

[46] Sigel K , Wisnivesky JP . Comorbidity profiles of patients with lung cancer: a new approach to risk stratification? Ann Am Thorac Soc. 2017;14(10):1512‐1513. 28961028 10.1513/AnnalsATS.201706-442ED

[47] de Groot V , Beckerman H , Lankhorst GJ , Bouter LM . How to measure comorbidity. A critical review of available methods. J Clin Epidemiol. 2003;56(3):221‐229. 12725876 10.1016/s0895-4356(02)00585-1

[48] People H. Social determinants of health. Washington, DC: US Department of Health and Human Services, Office of Disease Prevention, and Health Promotion https://www.healthypeople.gov/2020/topicsobjectives/topic/social-determinants-of-health Accessed May. 2020; 30:2017.

[49] Braveman P , Gottlieb L . The social determinants of health: it's time to consider the causes of the causes. Public Health Rep. 2014;129(2):19‐31. 10.1177/00333549141291S206 PMC3863696 24385661

